# Large effects on body mass index and insulin resistance of fat mass and obesity associated gene *(FTO) *variants in patients with polycystic ovary syndrome (PCOS)

**DOI:** 10.1186/1471-2350-11-12

**Published:** 2010-01-21

**Authors:** Susanne Tan, André Scherag, Onno Eilard Janssen, Susanne Hahn, Harald Lahner, Tiina Dietz, Susann Scherag, Harald Grallert, Carla Ivane Ganz Vogel, Rainer Kimmig, Thomas Illig, Klaus Mann, Johannes Hebebrand, Anke Hinney

**Affiliations:** 1 Department of Endocrinology and Division of Laboratory Research, University of Duisburg-Essen, Essen, Germany; 2 Institute for Medical Informatics, Biometry and Epidemiology, University of Duisburg-Essen, Essen, Germany; 3 Endokrinologikum Hamburg, Center for Endocrine and Metabolic Diseases, Hamburg, Germany; 4 Center for Endocrine and Metabolic Diseases, Wuppertal, Germany; 5 Department of Child and Adolescent Psychiatry, University of Duisburg-Essen, Essen, Germany; 6 Institute of Epidemiology, GSF - National Research Center for Environment and Health, Munich, Germany; 7 Department of Gynecology and Obstetrics, University of Duisburg-Essen, Essen, Germany

## Abstract

**Background:**

The polycystic ovary syndrome (PCOS), a common endocrine disorder in women of child-bearing age, mainly characterised by chronic anovulation and hyperandrogenism, is often associated with insulin resistance (IR) and obesity. Its etiology and the role of IR and obesity in PCOS are not fully understood. We examined the influence of validated genetic variants conferring susceptibility to obesity and/or type 2 diabetes mellitus (T2DM) on metabolic and PCOS-specific traits in patients with PCOS.

**Methods:**

We conducted an association study in 386 patients with PCOS (defined by the Rotterdam-criteria) using single nucleotide polymorphisms (SNPs) in or in proximity to the fat mass and obesity associated gene *(FTO*), insulin-induced gene-2 (*INSIG2*), transcription factor 7-like 2 gene (*TCF7L2*) and melanocortin 4 receptor gene (*MC4R*). To compare the effect of *FTO *obesity risk alleles on BMI in patients with PCOS to unselected females of the same age range we genotyped 1,971 females from the population-based KORA-S4 study (*Kooperative Gesundheitsforschung im Raum Augsburg*, Survey 4).

**Results:**

The *FTO *risk allele was associated with IR traits and measures of increased body weight. In addition, the *TCF7L2 *SNP was associated with body weight traits. For the SNPs in the vicinity of *INSIG2 *and *MC4R *and for the other examined phenotypes there was no evidence for an association. In PCOS the observed per risk allele effect of *FTO *intron 1 SNP rs9939609 on BMI was +1.56 kg/m^2^, whereas it was +0.46 kg/m^2 ^in females of the same age range from the general population as shown previously.

**Conclusion:**

The stronger effect on body weight of the *FTO *SNP in PCOS might well have implications for the etiology of the disease.

## Background

The polycystic ovary syndrome (PCOS) is a common endocrinopathy affecting about 6% of women of child-bearing age [1]. It is classically characterised by chronic anovulation, hyperandrogenism and polycystic ovarian morphology on ultrasonography [2]. In addition, a close relationship exists between obesity, insulin resistance (IR) and PCOS [3,4]. Despite a growing body of evidence demonstrating a substantial heritability of PCOS and the intrinsic impact of IR on the development of PCOS, its etiology and underlying pathophysiology still remains elusive.

To date, multiple genetic studies in PCOS have been performed examining genes coding for enzymes of steroid biosynthesis like CYP11, CYP17, CYP19, androgen receptor, insulin, insulin receptor and enzymes in the post-receptor signal cascade of insulin. However, a variant contributing substantially to the development of the PCOS phenotype was not detected [5-8]. Genome-wide association studies (*GWAS*) offer a new approach to gene discovery unbiased with regard to the presumed function of causal variants. Up to one million single nucleotide polymorphisms (SNP) that are distributed evenly across the whole genome are commonly used for this approach. In the field of human body weight regulation these studies already had a major impact on the identification of relevant polygenes. For instance, common variants in the fat mass and obesity associated gene (*FTO*) predispose to an elevated body mass index (BMI) with an increase of 0.36 kg/m^2 ^BMI units per risk allele. Homozygous risk allele carriers weighed about three kilograms more and had 1.67-fold higher odds for obesity compared to adults not inheriting the risk allele [9]. This robust association was also detectable in the first GWAS for early onset extreme obesity [10]. With regard to insulin sensitivity, a recent British study in healthy adults indicated an association between polymorphisms of the *FTO *gene and a decrease in insulin sensitivity, which was presumably BMI mediated [11]. Barber et al. additionally demonstrated an association between PCOS status and *FTO *obesity risk SNPs in a case-control study [12]. Using a similar approach, Attaoua et al. discussed the potential role of *FTO *variants for the glucose intolerance component of the metabolic syndrome in patients with PCOS [13]. Association with increased risk of obesity has also been demonstrated for SNP rs7566605 located ~10 kb upstream of the insulin-induced gene-2 (*INSIG2*), which was detected in the first population-based GWAS for body weight [14]. Results concerning the influence of this SNP on obesity risk are conflicting, as not all studies detected the association with obesity [15-18]. However, in a meta-analysis of case-control and family-based approaches comprising about 17,000 individuals a marginal effect of rs7566605 on obesity was still present [19]. Recently, Reinehr et al. showed a lower success rate of a lifestyle intervention for obese children homozygous for the risk allele C [20]. A solidly validated gene for type two diabetes mellitus (T2DM) is the transcription factor 7-like 2 gene (*TCF7L2*). Identified via a conventional genome wide linkage scan for T2DM and subsequent fine mapping [21], it had convincingly been confirmed in numerous studies including GWAS [22-24]. The gene codes for a stimulating regulator of proglucagon gene expression [25]. The proglucagon gene is post-translationally processed to produce GLP-1, a stimulator of insulin secretion and biosynthesis and it is an inhibitor of glucagon release [26]. GWAS reported association of a SNP in *TCF7L2 *with T2DM and IR in Caucasians [21,27,28] but a negative association to body weight [21,28-30]. In PCOS patients, using a sample size and design similar to their *FTO *study [12], a case-control comparison did not provide evidence for an association of *TCF7L2 *variants with disease status [31]. A further (negative) correlation to weight status was robustly found for a non-synonymous polymorphism Val103Ile in the melanocortin-4 receptor gene (*MC4R*) [32-34]. Interestingly, another genetic locus in the vicinity of the *MC4R *was recently also detected by a large-scaled GWAS [35]. The respective SNP was associated with obesity; it is located 188 downstream of the *MC4R*; its effect is presumably exerted by an influence on the expression of the gene. Additionally, another SNP near the previously described one, was shown to be associated with waist circumference and insulin resistance [36].

Given the high prevalence of obesity in PCOS and the compelling evidence for a substantial genetic background of both PCOS and obesity, known obesity susceptibility genes emerge as eligible candidates that might also be involved in the development of PCOS. Therefore we examined the influence of the described genetic variants on obesity and other endophenotypes in patients with PCOS. We considered polymorphisms in or near *FTO*, *INSIG2*, *TCF7L2 *and *MC4R *that are all well supported by large data collections of GWAS or meta-analyses for our analyses in patients with PCOS.

## Methods

### Patient Recruitment

The study was approved by the Ethics Committees of the University of Essen and Munich. All subjects gave written informed consent. The study was carried out according to the Declaration of Helsinki.

Consecutive, currently untreated patients with PCOS (n = 386) were recruited between 2/2001 and 1/2007 at the outpatient clinic of the Department of Endocrinology and Division of Laboratory Research, University of Duisburg-Essen, Germany. Some patients were also attracted by the PCOS homepage of the clinic http://www.pco-syndrom.de. PCOS was defined according to the 2003 Rotterdam criteria, so diagnosis of PCOS was established, if two of the three criteria chronic anovulation, clinical and/or biochemical hyperandrogenism and polycystic ovaries (PCO) were fulfilled and other pituitary, adrenal or ovarian diseases could be excluded [2].

### Clinical Characterization of Patients

Participants were carefully characterized with regard to medical history, clinical and socio-demographic variables using questionnaires, interview, and physical examinations, as previously described in detail [37]. Free androgen index (FAI) was calculated as total testosterone [nmol/l] × 100/SHBG [nmol/l]. Bioavailable testosterone concentration was calculated based on measured testosterone, SHBG and albumin levels [38]. Variables of IR and β-cell function were evaluated using a 3-h oral glucose tolerance test. After an overnight fast of 12 h patients ingested 75 g glucose and had their glucose and insulin levels determined at baseline and at 30, 60, 90, 120 and 180 min. IR was defined by the homeostasis model assessment (HOMA) model [39] and hyperinsulinemia by calculating the area under the curve of insulin response (AUCI).

### Females from the population-based sample (KORA-S4)

KORA (*Kooperative Gesundheitsforschung im Raum Augsburg*, Survey 4; 'Cooperative Health Research in the Region of Augsburg') is an epidemiological study group including 4,261 German adults representative of the population within the age range of 25-74 years in the city and region of Augsburg (Bavaria, Germany); probands were recruited between 1999-2001 [40]. *FTO *(rs9939609) genotypes as well as phenotype data were available for 1,971 females (mean BMI 26.96 ± 5.28 kg/m^2^, mean age 48.85 ± 13.69 years).

### Biochemical Analyses

Automated chemiluminescence immunoassay systems were used for the determination of LH, FSH, TSH, testosterone, estradiol, cortisol, free thyroxine, prolactin, blood glucose (ADVIA Centaur, Siemens, Germany), ACTH, dehydroepiandrosterone sulfate, androstenedione, sex hormone-binding globulin, insulin and insulin-like growth factor (IMMULITE 2000, Siemens, Germany). Measurement of blood glucose was performed by photometric determination (ADVIA 2400, Siemens, Germany). Intraassay variation was < 5% and interassay variation was < 8% for all measured variables. 17-hydroxyprogesterone was measured by the BIOSOURCE 17-alpha-OH-RIA-CT kit (Biosource International, California, USA) (analytical sensitivity 0.02 ng/ml) provided by IBL Hamburg (IBL, Gesellschaft für Immunchemie und Immunbiologie, Hamburg, Germany). The intra- and interassay coefficients of variation were 5.6 and 7.2% respectively. Except for amenorrhoic women, all laboratory variables were determined in the early follicular phase of the menstrual cycle.

### Genotyping

Genomic DNA was isolated from EDTA-anticoagulated blood using standard procedures. Genotyping of *FTO *SNP rs9939609 was performed by matrix-assisted laser desorption ionization-time of flight mass spectrometry (MALDI-TOF MS) analysis of allele-dependent primer extension products as described elsewhere [41]. For *MC4R *Val103Ile (rs2229616), DNA samples were genotyped as described previously. *MC4R *SNPs rs17782313 and rs12970134 were genotyped using TaqMan^® ^assays (Applied Biosystems, Foster City, CA, USA). All TaqMan^® ^probes and primers were purchased from Applied Biosystems (assays on demand; http://www.appliedbiosystems.com). For the *TCF7L2 *rs79031746, genotyping was performed by allele-specific PCR reactions (tetra-ARMS PCR; primers were as follows: (forward inner primer (C allele): 5'-TAGAGAGCTAAGCACTTTTTAGAGAC-3'; reverse inner primer (T allele): 5'-CTCATACGGCAATTAAATTATAGAA-3'; forward outer primer: 5'-AATTTTTTCACATGTGAAGACATAC-3'; reverse outer primer: 5'-AAGAGATGAAATGTAGCAGTGAAG-3'. The C allele is detected by a 202 bp amplicon and the T allele by a 272 bp amplicon. The product size of two outer primers is 424 bp.

Genotyping of *INSIG2 *SNP rs7566605 was carried out by PCR-RFLP with *Bsp*143I (digests the C-allele; primers: 5'-TGAAGTTGATCTAATGTTCTCTCTCC-3' and 5'-AAACCAAGGGAATCGAGAGC-3'). PCR products were run on ethidium bromide-stained 2.5% agarose gels. Positive controls for the variant alleles and a negative control (water) were run on each gel. To validate the genotypes, allele determinations were rated independently by at least two experienced individuals. Discrepancies were resolved unambiguously either by reaching consensus or by retyping. Missings were retyped.

### Statistical Analyses

All genotype distributions were tested for deviations from Hardy Weinberg equilibrium (HWE) using the PLINK software (Version 1.06 [42]) and no evidence for such deviations was detected (all p >> 0.4). All endophenotypes were analysed by either linear or logistic regression analyses using age in years as a covariate. Each SNP was analyzed under an (log-) additive genetic model, except for *MC4R *(rs2229616), where a dominant mode of inheritance was assumed. Nominal two-sided p-values, genetic effect sizes estimates and 95% confidence intervals (CI) for the estimates were derived. To address, the problem of testing multiple hypotheses, we also derived multiplicity-adjusted p-values for our strongest association signals (see Table three) using a method of described in [43]. As secondary analyses, robust linear regressions were used for the comparison of the PCOS patients with the population-based females and for an exploration of joint or interaction effects of *FTO *and *TCF7L2 *SNPs; model assumptions were checked graphically.

Power calculations were done with the software QUANTO (Version 1.2.3, http://hydra.usc.edu/gxe). 386 PCOS patients were estimated to yield a power > 0.80 to detect a standardized additive genetic effect size of 0.5 (α = 0.05; two-sided) assuming a minor allele frequency (MAF) of 5% and a standard normally distributed phenotype. For larger MAFs like 46.2%, smaller effect sizes of 0.2 will be detectable with a similar power. For comparison, standardized effect sizes like 0.1 have for example been reported for markers in FTO or TCF7L2 in unselected or control individuals for quantitative traits [9,44]. A standardized effect size of e.g. 0.1 in the additive genetic model implies that the distributions for the three genotypes are shifted by 1/10 of as standard deviation for the respective quantitative trait. Thus, except for rs2229616 with its low allele frequency (1.6%, Table 1), the study was well powered to detect strong to moderate genetic effects which might be present in our highly selected sample of PCOS patients.

**Table 1:** Characteristics of the investigated samples of the patients with PCOS (means and standard deviations or percent).

variables	386 patients with PCOS
age [years]	28.0 ± 6.5
body weight [kg]	88.3 ± 25.8
BMI [kg/m^2^]	31.4 ± 8.7
waist [cm]	98.8 ± 62.4
eumenorrhea [%]	8.7
oligomenorrhea [%]	65.7
amenorrhea [%]	25.5
hirsutism-Score [F/G]	8.9 ± 6.6
hirsutism [%]	54.0
acne [%]	47.8
alopecia [%]	27.3
PCO [%]	82.8
HA + OA [%]	92.3
HA + OA with PCO [%]	57.5
HA + OA without PCO [%]	12.2
HA + OA, PCO unknown [%]	30.3
HA + PCO [%]	5.2
OA + PCO [%]	2.5
testosterone [nmol/l]	2.8 ± 2.8
FAI	20.3 ± 83.9
bioavailable testosterone [ng/ml]	0.4 ± 0.8
HOMA-IR [μmol/lxmmol/l^2^]	3.7 ± 3.3
AUCI [mUxh/l]	262.6 ± 184.1
*FTO *(rs9939609; MAF A allele)	46.2
*INSIG2 *(rs7566605; MAF C allele)	33.8
*TCF7L2 *(rs7903146; MAF T allele)	28.4
*MC4R *(rs2229616; MAF T allele)	1.6
*MC4R *(rs17782313; MAF C allele)	29.0
*MC4R *(rs12970134; MAF A allele)	31.7

BMI = body mass index; F/G = hirsutism-score by Ferriman/Gallwey; FAI = free androgen index; PCO = polycystic ovary; HA = hyperandrogenism; OA = oligo-/amenorrhea; HOMA-IR = homeostasis model assessment for insulin resistance; AUCI = area under the curve of insulin response; MAF = minor allele frequency

## Results

The clinical and phenotypical characteristics of the investigated patients with PCOS as well as the estimated minor allele frequencies (MAFs) are given in Table 1. Table 1 also includes subgroup distributions according to the Rotterdam criteria (hyperandrogenism (HA), oligo-anovulation (OA) and polycystic ovaries (PCO)) as metabolic differences between theses subgroups have been described [45-48].

**Figure 1:**
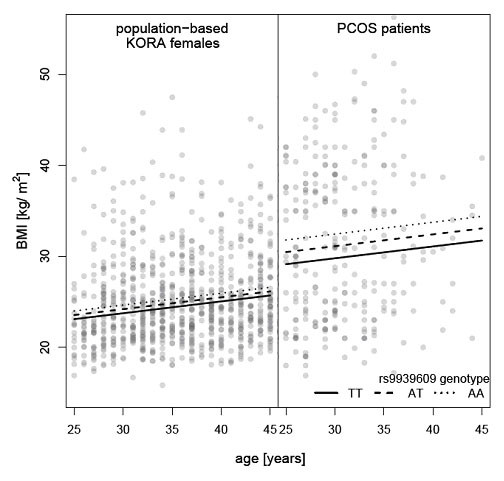
**Relationship between the *FTO *SNP rs9939609, BMI and age in females for the general population and in patients with PCOS**. Each dot represents one observation; the lines are derived from a robust linear regression on the complete data set of females with BMI as outcome and age (linear), group, genotype in the logadditive model and the interaction group x genotype as predictors.

Exploration of associations between genotypes and metabolic/obesity related variables in patients with PCOS revealed some evidence for an association of variants in *FTO *and *TCF7L2*, whereas no indication of association was observable for SNPs in *INSIG2 *and *MC4R *(see Table 2). We estimated that each risk allele of the polymorphism rs9939609 of *FTO *increased the body weight by ~4.6 kg, whereas the BMI was increased by ~1.6 kg/m^2 ^and the waist circumference increased by ~3.5 cm. Of note, the effect size of rs9939609 on BMI in PCOS was larger than in unselected females from the general population in the same age range (see Figure 1). For the *TCF7L2 *polymorphism rs7903146 the per risk allele effect was ~4.2 kg body weight, ~1.4 kg/m^2 ^BMI and ~3.4 cm waist circumference, respectively (for details see Table 2). There was no evidence for a potentially strong interaction or a correlation between the *FTO *(adjusted effect 1.35 kg/m^2 ^in robust linear regression) and the *TCF7L2 *locus (adjusted effect 1.45 kg/m^2^). Moreover, *FTO *variant rs9939609 was associated with variables of insulin resistance (see Table 3). As an example, the estimated per risk allele effect was 3.25 mU/l for fasting insulin, which changed to 1.88 mU/l (95%CI 0.22 mU/l; 3.54 mU/l, p-value = 0.03), if BMI was added as a covariate to the regression model. Thus, even after correcting for BMI, a genetic effect of rs9939609 on fasting insulin levels was observed. By contrast, there was no evidence for an association of SNPs in *INSIG2, TCF7L2 *and *MC4R *with variables of insulin resistance (for details see Table 3).

**Table 2:** Association analysis for the relationship between validated SNPs in the explored candidate genes and quantitative metabolic/obesity related variables in PCOS with genetic effect sizes estimates derived for an additive genetic (*MC4R *rs2229616 dominant genetic model) for the minor allele (see also Table 1).

	**body weight [kg]**			**BMI [kg/m^2^]**			**waist [cm]**		
			
**gene (SNP) minor allele**	**β**	**95% CI**	**p**	**β**	**95% CI**	**p**	**β**	**95% CI**	**p**
			
*FTO *(rs9939609) A	4.60	(0.97;8.22)	0.01	1.56	(0.34;2.78)	0.01	3.49	(0.62;6.37)	0.02
									
*INSIG2 *(rs7566605) C	-1.38	(-5.23;2.48)	0.48	-0.35	(-1.66;0.95)	0.59	-1.89	(-4.92;1.15)	0.22
									
*TCF7L2 *(rs7903146) T	-4.18	(-8.20;-0.15)	0.04	-1.35	(-2.71;0.01)	0.05	-3.43	(-6.63;-0,24)	0.04
									
*MC4R *(rs2229616) T	-2.12	(-16.88;12.63)	0.78	-0.91	(-5.88;4.06)	0.72	1.14	(-10.01;12.29)	0.84
									
*MC4R *(rs17782313) C	0.33	(-3.75;4.42)	0.87	-0.01	(-1.36;1.39)	0.98	0.54	(-2.73;3.81)	0.75
									
*MC4R *(rs12970134) A	0.20	(-3.74;4.14)	0.92	-0.12	(-1.45;1.21)	0.86	0.52	(-2,64;3.69)	0.75

SNP = single nucleotide polymorphism; BMI = body mass index

**Table 3:** Association analysis for the relationship between validated SNPs in the explored candidate genes and quantitative variables of insulin resistance in PCOS with genetic effect sizes estimates derived for an additive genetic model (*MC4R *rs2229616 dominant genetic model) for the minor allele (see also Table 1).

	**fasting insulin [mU/l]**			**HOMA-IR [μmol/lxmmol/l^2^]**			**AUCI [mUxh/l]**		
			
**gene (SNP) minor allele**	**β**	**95% CI**	**p**	**β**	**95% CI**	**p**	**β**	**95% CI**	**p**
			
*FTO *(rs9939609) A	3.25	(1.32;5.18)	0.001*	0.76	(0.29;1.23)	0.002*	35.92	(9.63;62.21)	0.01
									
*INSIG2 *(rs7566605) C	0.85	(-1.21;2.90)	0.42	0.33	(-0.18;0.83)	0.21	12.92	(-14.83;40.68)	0.36
									
*TCF7L2 *(rs7903146) T	-0.23	(-2.40;1.93)	0.83	-0.13	(-0.66;0.40)	0.63	-5.18	(-34.27;23.91)	0.73
									
*MC4R *(rs2229616) T	3.20	(-4.67;11.07)	0.43	-0.46	(-2.45;1.53)	0.65	-8.25	(-117.9;101.4)	0.88
									
*MC4R *(rs17782313) C	1.10	(-1.08;3.28)	0.32	0.17	(-0.36;0.70)	0.54	4.52	(-25.02;34.06)	0.76
									
*MC4R *(rs12970134) A	1.07	(-1.03;3.18)	0.32	0.20	(-0.31;0.71)	0.45	1.23	(-27.21;29.68)	0.93

SNP = single nucleotide polymorphism; HOMA-IR = homeostasis model assessment for insulin resistance; AUCI = area under the curve of insulin response

*both associations would have been "significant" at the alpha = 0.10 level if correction for multiple testing would have been applied resulting in adjusted p-values of 0.062 for rs9939609 and fasting insulin and 0.099 for rs9939609 and HOMA-IR

Finally, we investigated the relationship between the variants and PCOS specific characteristics (for details see Tables 4 and 5). There was no association to chronic anovulation, hirsutism, acne, alopecia or androgen levels for any of the variants. Interestingly, we observed that the obesity risk allele A of the *FTO *variant rs9939609 was less frequently observed in patients with polycystic ovarian morphology (PCO) compared to PCOS patients without PCO (OR = 0.57, 95%, CI 0.36;0.88, p-value = 0.01) and even after correction for BMI this relationship was still present (OR = 0.60, 95%CI 0.37;0.93, p-value = 0.02).

**Table 4:** Association analysis for the relationship between validated SNPs in the explored candidate genes and PCOS symptoms (present/absent) with genetic effect sizes estimates derived for an log-additive genetic model (*MC4R *rs2229616 dominant genetic model) for the minor allele (see also Table 1).

	**chronic anovulation**			**hirsutism-score [F/G] > 7 *hirsutism-score [F/G]***			**alopecia**			**acne**			**PCO**		
					
**gene (SNP) minor allele**	**OR**	**95% CI**	**p**	**OR *β***	**95% CI**	**p**	**OR**	**95% CI**	**p**	**OR**	**95% CI**	**p**	**OR**	**95% CI**	**p**
					
*FTO *(rs9939609) A	1.29	(0.70;2.37)	0.41	1.15 *0.34*	(0.87;1.54) *(0,59;1.28)*	0.33 *0.47*	0.90	(0.65;1.24)	0.50	0.97	(0.73;1.30)	0.85	0.57	(0.36;0.88)	0.01
															
*INSIG2 *(rs7566605) C	1.02	(0.54;1.90)	0.96	1.03 *-0.08*	(0.76;1.38) *(1.06;0.90)*	0.87 *0.87*	1.17	(0.83;1.64)	0.37	1.00	(0.74;1.35)	0.99	1.02	(0.65;1.60)	0.93
															
*TCF7L2 *(rs7903146) T	0.64	(0.35;1.18)	0.15	0.88 *0.09*	(0.64;1.21) *(0.95;1.12)*	0.44 *0.87*	0.75	(0.51;1.08)	0.12	0.84	(0.61;1.15)	0.28	0.77	(0.48;1.21)	0.25
															
*MC4R *(rs2229616) T	-	-	-	1.73 *-0.39*	(0.51;5.84) *(4.16;3.38)*	0.38 *0.84*	0.91	(0.24;3.46)	0.89	0.54	(0.16;1.82)	0.32	1.48	(0.18;12.50)	0.71
															
*MC4R *(rs17782313) C	1.01	(0.52;1.95)	0.98	0.85 *0.16*	(0.62;1.17) *(0.88;1.21)*	0.33 *0.76*	1.01	(0.70;1.44)	0.98	1.04	(0.76;1.43)	0.81	0.95	(0.59;1.52)	0.82
															
*MC4R *(rs12970134) A	0.74	(0.39;1.39)	0.34	0.97 *0.40*	(0.71;1.32) *(0.60;1.41)*	0.83 *0.43*	1.09	(0.77;1.54)	0.62	1.06	(0.78;1.45)	0.70	1.03	(0.64;1.64)	0.71

SNP = single nucleotide polymorphism; F/G = hirsutism-score by Ferriman/Gallwey

Note that the hirsutism-score was also evaluated quantitatively similar to the other quantitative measures (in italics).

**Table 5:** Association analysis for the relationship between validated SNPs in the explored candidate genes and quantitative **variables of hyperandrogenemia **in PCOS with genetic effect sizes estimates derived for a log-additive genetic model (*MC4R *rs2229616 dominant genetic model) for the minor allele (see also Table 1).

	**testosterone [nmol/l]**			**FAI**			**bioavailable testosterone [ng/ml]**		
			
**gene (SNP) minor allele**	**β**	**95% CI**	**p**	**β**	**95% CI**	**p**	**β**	**95% CI**	**p**
			
*FTO *(rs9939609) A	-0.01	(-0.41;0.39)	0.95	5.25	(-7.11;17.60)	0.41	0.03	(-0.09;0.15)	0.64
									
*INSIG2 *(rs7566605) C	-0.16	(-0.58;0.26)	0.46	-6.33	(-19.36;6.71)	0.34	-0.07	(-0.20;0.05)	0.25
									
*TCF7L2 *(rs7903146) T	0.20	(-0.25;0.64)	0.38	7.93	(-5.90;21.77)	0.26	0.04	(-0.09;0.18)	0.50
									
*MC4R *(rs2229616) T	-0.28	(-1.88;1.32)	0.73	-12.18	(-60.54;36.17)	0.62	-0.08	(-0.54;0.38)	0.73
									
*MC4R *(rs17782313) C	-0.25	(-0.70;0.19)	0.27	-3.47	(-17.33;10.40)	0.62	-0.05	(-0.18;0.08)	0.46
									
*MC4R *(rs12970134) A	0.15	(-0.29;0.58)	0.51	7.26	(-5.97;20.49)	0.28	0.07	(-0.06;0.19)	0.30

SNP = single nucleotide polymorphism; FAI = free androgen index

## Discussion

The influence of *FTO *intron 1 variation on body weight related phenotypes in patients with PCOS is underscored. In population-based GWAS the estimated per risk allele effect ranged between 0.36 kg/m^2 ^[9] and 0.66 kg/m^2 ^[49] for statistical models with a slightly different set of covariates. In our PCOS sample, we estimated an average effect of the *FTO *risk allele of 1.56 kg/m^2 ^(95%CI 0.34 kg/m^2^; 2.78 kg/m^2^) including age as a quantitative covariate. Thus, the impact of the *FTO *variants on BMI is larger in PCOS patients than in the general population with an average effect of the *FTO *risk allele of 0.46 kg/m^2 ^(95%CI 0.17 kg/m^2^; 0.75 kg/m^2^) as shown in Figure 1. Of note, our finding is surprisingly similar to those of other studies [12,50]. Barber et al. report a per risk allele effect of 1.1 kg/m^2 ^(95%CI -0.9 kg/m^2^; 3.2 kg/m^2^) in their PCOS cases and only 0.5 kg/m^2 ^(95%CI -0.1 kg/m^2^; 1.3 kg/m^2^) in their controls [12]. Moreover, this study is, to our knowledge, the first to describe a correlation between *FTO *rs9939609 and insulin resistance or hyperinsulinemia in PCOS patients reflected in fasting insulin, HOMA-IR as well as in AUCI levels After strictly controlling for the multiple statistical tests performed here (multiple phenotypes and multiple SNPs) the adjusted p-value for *FTO *rs9939609 and fasting insulin was 0.062 and the related effect, though weaker, was still present even after statistically correcting for BMI. Other study groups could not demonstrate an association between *FTO *and IR in obese and T2DM patients, respectively [51,52]. In PCOS, *FTO *variation seems to be a key marker for IR, either directly i.e. to some degree independent of BMI/obesity or secondary due to its impact on body weight which in turn has an impact on glucose intolerance and diabetes. Barber et al. at first demonstrated an association between *FTO *and PCOS status and hypothesized a mediation of this association by adiposity. However, in this British sample, no IR data were available. In a French sample *FTO *was associated with both glucose intolerance and metabolic syndrome [13] without finding an association to IR. The greater effect on BMI and association to IR in PCOS possibly reflects a pathogenetic function of the *FTO *gene in the development of PCOS. This study and the data from Barber et al. demonstrated, that the *FTO *gene does not influence any of the PCOS defining traits neither directly nor indirectly via an effect on obesity or IR. In this German cohort, it is even less frequently associated with PCO. In case of a pathogenetic role of *FTO *in PCOS, it seems to be limited to determine only the metabolic phenotype.

As second strongest signal, we found association between the T2DM susceptibility gene *TCF7L2 *risk allele and obesity related traits in patients with PCOS. The *TCF7L2 *obesity association was stochastically independent of the *FTO *association if assessed in a multiple regression analysis. While some did not find an influence on body weight [29,30,53-56], others have reported analogous associations of *TCF7L2 *and obesity related traits [28,43,57]. Despite possible effects related to differences in ascertainment schemes, multiple explanations for this conflicting findings are possible [57]. One possible explanation by Cauchi and collegues [43] is that the risk for T2DM related to *TCF7L2 *seems to be modulated by the obesity status of an individual with stronger effects in the obese. It is possible that our study picked-up this BMI-related effect of *TCF7L2*. As we observed no evidence for an association of *TCF7L2 *with IR or other PCOS specific symptoms, common variants of *TCF7L2 *most likely influence T2DM susceptibility through impairment of insulin secretion rather than IR [56,58]. Interestingly, this observation is consistent with the PCOS data of Barber et al. [12] who failed to detect association of *TCF7L2 *variation but who do not report on the genotype dependent BMI-distribution.

Finally, our data on rs7566605 located ~10 kb upstream of *INSIG2 *and on markers in proximity to *MC4R *provided no evidence for an involvement in the development of PCOS or related endophenotypes.

## Conclusion

In summary, we explored (mainly) GWAS-derived candidate gene markers for obesity and T2DM in patients with PCOS. We show that the impact of *INSIG2*, *TCF7L2 *and *MC4R *SNPs on multiple endophenotypes might either be rather weak or not present at all. For genetic variation in intron 1 of *FTO *we provide evidence for associations to metabolic aspects of PCOS with some emphasis on IR. Interestingly, the estimated effect on obesity seems to be larger than the effect derived from population-based samples. This stronger effect might well have implications for the etiology of the disease requiring focussed functional studies.

## Competing interests

The authors declare that they have no competing interests.

## Authors' contributions

ST contributed substantially to conception and design of the study, acquisition of data, analysis and interpretation of data and drafted the manuscript. AS contributed substantially to conception and design of the study, analysis of data, performed the statistical analysis and drafted the manuscript. OEJ, SH, HL, TD, RK and KM contributed to acquisition of data and revised the manuscript critically for important intellectual content. SS and CIGV carried out the molecular genetic studies for polymorphisms of *TCF7L2*, *INSIG2 *and *MC4R *and revised the manuscript critically for important intellectual content. HG and TI contributed to acquisition of data, carried out the molecular genetic studies for polymorphisms of *FTO *and revised the manuscript critically for important intellectual content. JH and AH contributed substantially to conception and design of the study, analysis and interpretation of data and were involved in drafting the manuscript and haves given final approval of the version to be published. All authors read and approved the final manuscript.

## Pre-publication history

The pre-publication history for this paper can be accessed here:

http://www.biomedcentral.com/1471-2350/11/12/prepub
